# Integrated Gas Chromatograph-Mass Spectrometry (GC/MS) and MS/MS-Based Molecular Networking Reveals the Analgesic and Anti-Inflammatory Phenotypes of the Sea Slater *Ligia exotica*

**DOI:** 10.3390/md17070395

**Published:** 2019-07-04

**Authors:** Yang Yue, Quanbin Zhang, Jing Wang

**Affiliations:** 1 Key Laboratory of Experimental Marine Biology, Institute of Oceanology, Chinese Academy of Sciences, 7 Nanhai Road, Qingdao 266071, China; 2 Laboratory for Marine Biology and Biotechnology, Qingdao National Laboratory for Marine Science and Technology, Qingdao 266237, China; 3 Center for Ocean Mega-Science, Chinese Academy of Sciences, 7 Nanhai Road, Qingdao 266071, China

**Keywords:** *Ligia* sp., analgesic effects, anti-inflammatory activities, molecular networking, GC-MS

## Abstract

The sea slater *Ligia exotica* is believed to have effects of reducing swelling and relieving pain in Chinese folk medicine. However, the scientific foundation of using the sea slater *Ligia* spp. as an analgesic and anti-inflammatory material remains elusive. In the present study, various organic extracts from sea slater *L. exotica* were subjected to biological screening employing in vitro and in vivo models, and chemical phenotypes of the biologically active extract were deciphered by integrated gas chromatograph-mass spectrometry (GC-MS) profiling and MS/MS-based molecular networking. The results demonstrated, for the first time, that petroleum ether extract (PE) from *L. exotica* possessed remarkable anti-inflammatory and analgesic effects. Moreover, intragastric administration of PE at 200 mg/kg produced analgesic effects in both the writhing test and hot plate test. GC-MS analysis revealed that Z-9-hexadecenoic acid and 6-octadecenoic acid dominated in the volatile compositions of PE. Molecular networking (MN) suggested great chemical diversity within *L. exotica*. In total, 69 known compounds were identified in *Ligia* extracts by MS/MS spectral matching, and at least 7 analogues from two clusters of nitrogen-containing compounds (MN_3,4_) were strongly suggested as novel compounds. The molecular families MN_1,3,4_ were almost exclusively detected in the biologically active PE and ethyl acetate extract (EE). Importantly, various known compounds identified in MN_1_ were reported to possess analgesic and anti-inflammatory effects in the literature, which may contribute to the observed analgesic and anti-inflammatory effects of *L. exotica*. The present study not only demonstrated the ethnopharmaceutical value of *L. exotica* for pain-relief in Chinese folk medicine, but also suggested that sea slaters may represent a promising source for discovery of novel analgesic and anti-inflammatory compounds in the near future.

## 1. Introduction

Pain is defined as an unpleasant sensory and emotional experience associated with actual or potential tissue damage, or described in terms of such damage, according to the International Association for the Study of Pain (IASP). It is now well accepted that pain is not only a symptom of many debilitating conditions, but the pain itself, especially chronic pain, is also a disease. It is estimated that there were 126 million adults in the United States experiencing varying degrees of pain in 2012, of which 17.6% experienced moderate to severe pain [1]. In Europe, the populations suffering from moderate to severe pain were up to 19% [2]. The prevailing occurrence of pain not only impairs the life quality of patients, but also brings high social and economic costs [3]. In 2010, the cost for pain relief in the United States reached 560–635 billion dollars, which was far higher than those of many serious diseases, such as heart disease (309 billion dollars), cancer (243 billion dollars), and diabetes (188 billion dollars) [4]. Clinically, opioid analgesics and non-steroidal anti-inflammatory drugs are the most commonly used drugs for pain relief. However, long-term application of these drugs can produce serious side effects [5,6]. Due to the complexity of pain mechanisms and adverse effects of the existing analgesics, there is still an unmet need for safely and effectively managing severe pain [7]. Therefore, searching for novel compounds with antinociceptive potential is currently an urgent task for the scientific community.

Marine creatures have proven to be a treasure for discovery of analgesic and anti-inflammatory lead compounds with structural novelty [8]. In the ocean, various marine algae and invertebrates are capable of synthesizing analgesic and anti-inflammatory metabolites, including a variety of terpenoids [9], alkaloids [10], glycosides [11], and peptides [12]. In 2004, United States Food and Drug Administration approved the first marine analgesic drug ziconotide (Prialt^®^) for severe pain and chronic pain [13], which was initially found in cone snail venom as highly selective n-type calcium channel Ca_v_2.2 inhibitors.

Sea slaters of the genus *Ligia*, also known as rock lice or sea louse, belong to the subphylum Crustacea of the Isopoda order and Ligiidae family and are widely distributed in coastal China [14]. Sea slaters are intertidal species that mainly occur in intertidal rocky shores and play important roles in the intertidal ecosystem. Importantly, the genus *Ligia* was considered to be a prototypal form of members of terrestrial Oniscidea, which successfully evolved from ancestral marine isopods to a fully land-adapted form [15]. This unique biological feature makes sea slaters a good example of evolution and fascinating to many biological scientists. Currently, about 43 species are included in the genus *Ligia*, and only two species, *Ligia exotica* Roux 1828 and *Ligia cinerascens* Budde-Lund 1885 have been found to occur along the Chinese coastal area.

The medicinal value of sea slaters has been recorded in Chinese Marine Materia Medica [16], a modern pharmaceutical dictionary covering Chinese traditional herbal, animal, and mineral medicine from marine sources. However, the pharmaceutical value of sea slaters was not recorded in the classic Compendium of Materia Medica, which was compiled by Li Shizhen of the Ming dynasty in 1578. The sea slaters were believed to have the effects of reducing swelling and relieving pain in Chinese folk medicine. Therefore, they were traditionally used by indigenous people in southern Chinese coastal areas to treat traumatic injuries with remarkable efficiency. However, the effector molecules within the sea slaters responsible for the pain-relief effects remain largely unknown.

There are only a handful of scientific publications reporting the chemical components and biological activities of extracts from sea slaters, which hampers insights into the biologically active molecules. Extracts from sea slaters were reported to have antitumor activity [17] and antimicrobial activity [18]. However, the chemical diversity of these extracts is not fully understood. Previous studies demonstrated that sea slaters *L. exotica* were able to produce a new inosine disaccharide [19]. Moreover, several carotenoids were detected in the adults, the eggs, and the larvae of *L. exotica* [20]. However, whether those compounds contribute to the pain-relief effects of sea slaters remains uninvestigated.

In the course of mining new marine resources with medical relevance, our attention is drawn to sea slaters, which have great potential in folk medicine. Therefore, the aim of the present study was to reveal the chemical basis underlying the analgesic and anti-inflammatory effects of extracts from the sea slater *L. exotica* by integrating gas chromatograph-mass spectrometry (GC-MS) profiling and MS/MS-based molecular networking.

## 2. Results

### 2.1. Solvent Extration and General Analysis of Ligia Extracts

**Figure 1:**
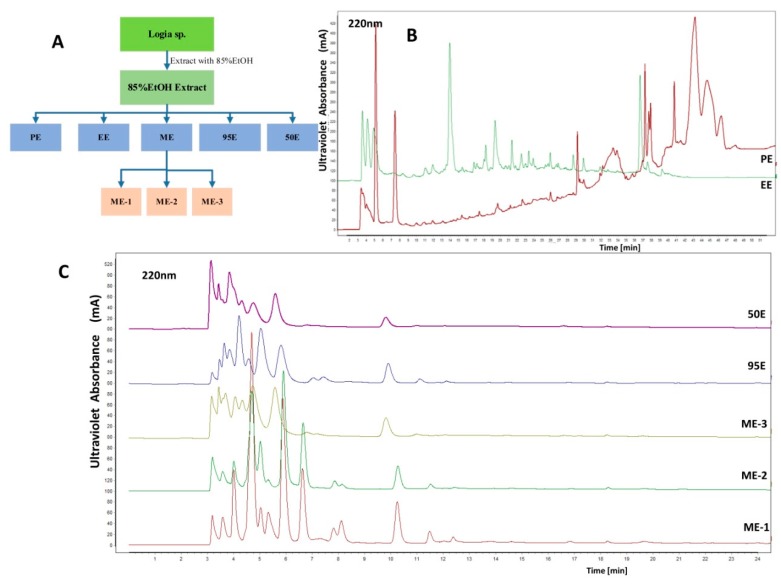
(**A**) Schematic illustration of the extraction process from fresh *L. exotica* with increasing polarity of organic solvents. (**B**,**C**) High performance liquid chromatography (HPLC) profiling of the *Ligia* extracts, petroleum ether extract (PE), ethyl acetate extract (EE), methanol extracts (ME-1, ME-2, ME-3), 95% EtOH extract (95E), and 50% EtOH extract (50E), using a C18 reversed phase column.

Freshly collected specimens of *L. exotica* were extracted with 85% ethanol (EtOH), and the resulting 85% EtOH extract was subject to successive extraction with petroleum ether, ethyl acetate, methanol, and EtOH, resulting in petroleum ether extract (PE), ethyl acetate extract (EE), methanol extract (ME; including ME-1, ME-2, ME-3), 95% EtOH extract (95E), and 50% EtOH extract (50E) (Figure 1A). Then, the chemical diversity of the seven organic extracts was characterized by high performance liquid chromatography (HPLC). PE and EE were more complex and lipophilic than other extracts (Figure 1B,C). Peaks occurred on the HPLC chromatograms of PE and EE had a diverse ultraviolet absorbance pattern in the range of 200–300 nm (Figure S1). ME, 95E, and 50E were preliminarily assumed to be largely composed of polar compositions, considering their poor retention in the reversed phase column and the solubility in water. Table 1 shows the yield of 85% EtOH, PE, EE, ME-1, ME-2, ME-3, 95E, and 50E from fresh *L. exotica* samples. The results suggest that the lipophilic PE and EE only account for approximate 15.7% in the 85% EtOH extract and 1.3% in the wet weight of fresh *L. exotica*, which indicates large proportions of hydrophilic compositions in the 85% EtOH extract.

### 2.2. Biological Activities in Vitro and in Vivo

**Figure 2:**
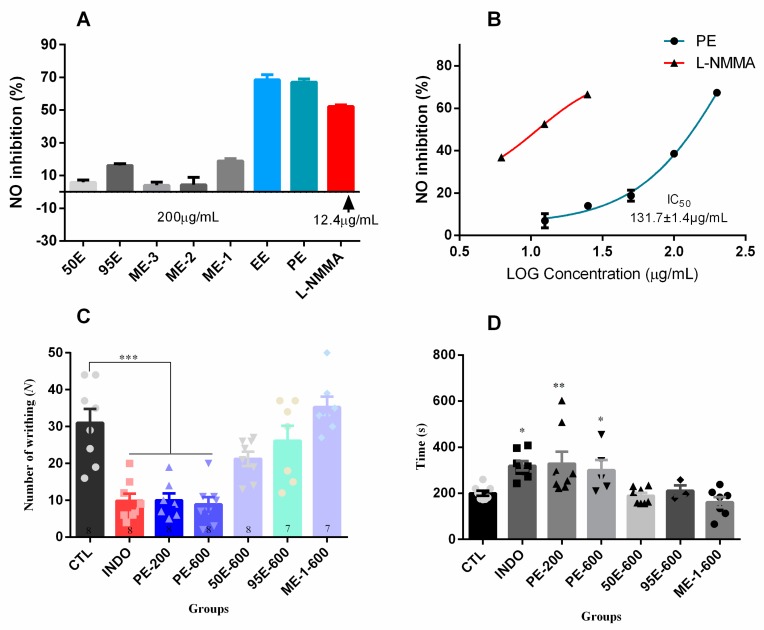
The inhibitory effects of various *Ligia* extracts, PE, EE, ME-1, ME-2, ME-3, 95E, and 50E on (**A**) the production of nitric oxide (NO) in lipopolysaccharide (LPS)-induced RAW264.7 macrophages. (**B**) The concentration-response inhibition curve of PE on NO production in LPS-induced RAW264.7 macrophages. *N*-monomethyl-l-arginine (L-NMMA) was taken as positive control. (**C**) The analgesic effects of PE, ME-1, 95E, and 50E in acetic-acid-induced writhing test; the number of writhing events was recorded within 25 min after intragastric administration (ig) of the *Ligia* extracts. (**D**) The time (s) of the first writhing response in mice within 25 min. Note: CTL = blank control; INDO = indomethacin (20 mg/kg); PE-200 = PE (200 mg/kg); PE-600 = PE (600 mg/kg); 50E-600 = 50E (600 mg/kg); 95E-600 = 95E (600 mg/kg); ME-1-600 = ME-1 (600 mg/kg); * *p* < 0.05, ** *p* < 0.05, *** *p* < 0.001 are considered significantly different compared to the control group CTL.

Biological activities of the *Ligia* extracts were assayed in vitro and in vivo employing various biological phenotypes. Anti-inflammatory effects of the *Ligia* extracts were investigated by measuring nitric oxide (NO) production in lipopolysaccharide (LPS)-induced RAW264.7 macrophages. We found that PE and EE exhibited the highest inhibitory effects among all the *Ligia* extracts, causing approximately 70% inhibition at 200 μg/mL (Figure 2A). However, the inhibition rates of ME-1, ME-2, ME-3, 95E, and 50E were below 20% at 200 μg/mL. The half maximal inhibitory concentration (IC_50_) value of PE on NO inhibition was 131.7 ± 1.4 μg/mL (*n* = 3) (Figure 2B).

The analgesic activities of *L. exotica* were validated by acetic-acid-induced abdominal constriction test and hot-plate test (Figure 2C,D and Table 2). In the acetic-acid-induced writhing test, low dosage of PE (200 mg/kg, ig) significantly reduced the number of writhing instances from 28.0 ± 4.3 (mean ± S.E.M, *n* = 8) to 10.0 ± 1.9 (*n* = 8, *p* < 0.001), which was comparable to indomethacin (INDO, 20 mg/kg) (Figure 2C). The number of writhing instances induced by high dosage of PE (600 mg/kg, ig) was 8.8 ± 2.0 (*n* = 8), which was significantly lower than that of the control group. Moreover, administration of PE either at 200 mg/kg or 600 mg/kg significantly prolonged the time of the first writhing response (Figure 2D). However, ME-1, 95E, and 50E did not exhibit significant analgesic effects, even in high doses (600 mg/kg, ig) (Figure 2C,D). The hot-plate test was further performed to confirm the analgesic effect of PE. The results suggest that PE significantly increases the latency time in a dose-dependent manner within 2 h after intragastric administration. More importantly, high dosage of PE (600 mg/kg, ig) could increase the heat tolerance in mice from 16.67 ± 1.19 s (*n* = 8–10) to 28.61 ± 2.41 s (*n* = 8–10) (Table 2).

**Figure 3:**
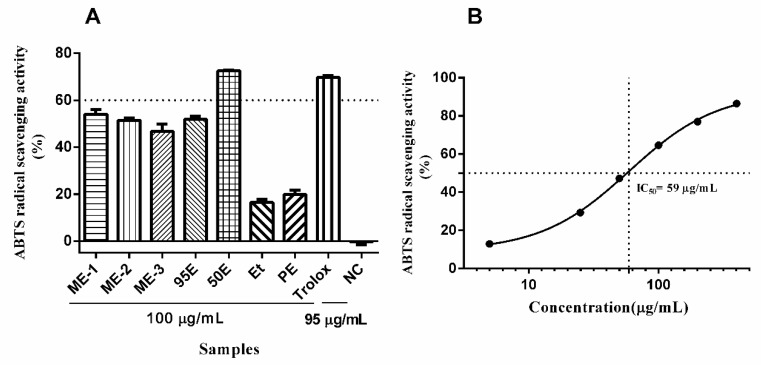
(**A**) The ABTS radical scavenging activities of *Ligia* extracts PE, EE, ME-1, ME-2, ME-3, 95E, and 50E, assayed at the final concentrations of 100 μg/mL using Total Antioxidant Capacity Assay Kit. Trolox (final concentration, 95 μg/mL) was used as positive control. Note: NC = negative control. (**B**) The antioxidant activities of 50E assayed with increasing concentrations from 5 μg/mL to 400 μg/mL. Results are expressed as means ± S.E.M. (*n* = 3).

In addition, the antioxidant activities of *Ligia* extracts were also assayed using 2,2′-azino-bis(3-ethylbenzthiazoline-6-sulfonic acid (ABTS) method. As illustrated in Figure 3A, of all the *Ligia* extracts, only 50E exhibited potent antioxidant activity (>60% inhibition) at 100 μg/mL, which was comparable to the positive control Trolox (95 μg/mL). Of note, both PE and EE possessed weak antioxidant activities. The IC_50_ value of 50E was determined to be about 59 μg/mL (Figure 3B).

### 2.3. Identification of the Lipophilic PE and EE by Gas Chromatograph-Mass Spectrometry (GC-MS)

**Figure 4:**
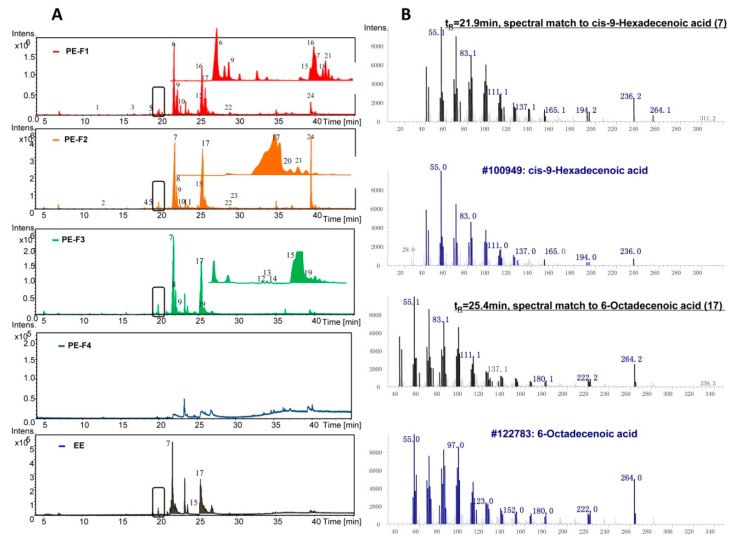
(**A**) The gas chromatograph-mass spectrometry (GC-MS) chromatograms of PE and EE. The lipophilic fractions of PE, Fraction(Fr.)1-3, and EE were subject to GC-MS profiling and the resulting MS spectra were compared with the reference compounds in the NIST08 database. The peak number in Figure 4A indicates the putative identification of known compounds with the matching degree ≥ 95%. (**B**) Two major identified compounds matching to Z-9-hexadecenoic acid and 6-octadecenoic acid with a matching degree of 99% at t_R_ = 21.9 min and t_R_ = 25.4 min in PE and EE. The black boxes in Figure 4A indicate unassigned compounds in the NIST08 database.

The volatile compounds in the biologically active PE and EE were identified by GC-MS profiling. To remove the polar components in PE, silica gel column chromatography was used. PE was then fractioned into 5 parts Fraction(Fr.)1-5 using petroleum ether-ethyl acetate and dichloromethane-methanol solvent system (Figure S2). Then, fractions Fr.1-4 and EE were subject to GC-MS analyses. In general, GC-MS chromatograms of the PE fractions Fr.1-3 and EE were very similar, which differed from the fraction Fr.4 (Figure 4A). By comparison with known compounds in the National Institute of Standards and Technology (NIST) database, there were 24 compounds identified from PE and EE with a matching degree cutoff value of 95% (Table 3). Of all the identified volatile compounds, unsaturated fatty acids and their esters dominated. Most identified compounds eluted at t_R_ = 21.0–26.0 min. As an obvious feature in the chromatograms of PE and EE, two major peaks at t_R_ = 21.9 min and t_R_ = 25.4 min matched to Z-9-hexadecenoic acid (7, palmitoleic acid) and 6-octadecenoic acid (17, petroselinic acid), respectively (Figure 4B, Table 3).

Table S1 shows the major volatile compounds in EE and PE. Z-9-hexadecenoic acid, Z-11-hexadecenoic acid, E-13-octadecenoic acid, and 6-octadecenoic acid dominated in the fraction Fr.1-3 of PE. However, only Z-9-hexadecenoic acid was detected as a major compound in EE (Table S1). Moreover, unsaturated fatty acids accounted for a large proportion of the identified 24 compounds, up to 55–77% in fraction Fr.1-3 and 44% in EE (Table S1). In addition, the highest amounts of cholesterol were detected in fraction Fr.2 of PE (Figure 4A, Table S1). Of note, there were still about 20% unidentified peaks in the GC-MS chromatograms of PE Fr.1-3, while the proportion of unidentified peaks in EE was as high as 46% (Table S1).

### 2.4. HPLC-MS/MS Analysis of the Ligia Extracts and Dereplication by Molecular Networking

**Figure 5:**
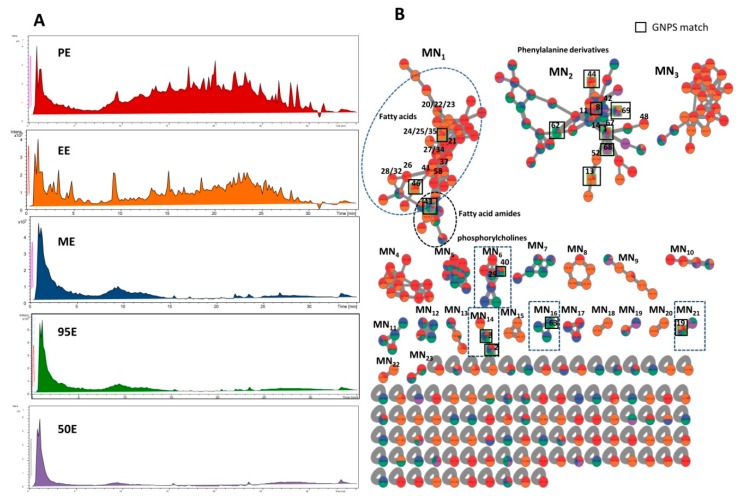
(**A**) Total ion chromatograms (TICs) of PE, EE, ME, 95E, and 50E. (**B**) MS/MS-based molecular network of five organic extracts from *L. exotica*. The molecular network was created using the online Global Natural Products Social (GNPS) workflow with a cosine score cutoff value of 0.70. The color of the nodes informs the source of the precursor ions, and the edge thickness indicates the cosine score, which is closely related to the structural similarity. The nodes in red correspond to compounds present in PE; nodes in orange correspond to compounds present in the EE; nodes in dark blue correspond to compounds in ME; nodes in green correspond to compounds present in the 95E; and nodes in purple correspond to compounds from 50E. The GNPS spectral matches in MN_1–23_ are suggested by black squares.

To understand the biological phenotypes of PE observed in the analgesic and anti-inflammatory experiments, *Ligia* extracts were subject to HPLC-qTOF-ESI-MS/MS analysis. The total ion chromatograms (TICs) of PE, EE, ME, 95E, and 50E are presented in Figure 5A. In accordance with the HPLC chromatograms, PE and EE possessed the most complex compositions compared with ME, 95E, and 50E. To visualize the global chemical phenotypes in these extracts, MS/MS-based molecular networking (MN) was established by using the online Global Natural Products Social (GNPS) MN platform. The comprehensive molecular networks of the five extracts consisted of 288 nodes and 191 paired nodes, which were grouped into 23 sub-MNs interconnected with 375 edges after removal of non-clustered nodes (Figure 5B). Among the 288 nodes, only 17 nodes were annotated by GNPS MS/MS spectral library matching, and the matching hits are presented in Figure 5B and Table 4. Two major sub-MNs, namely MN_1_,_2_, composed of more than 40 nodes, were easily distinguished. Of 43 nodes, only 3 hits—8-HETE (35), (Z)-9-octadecenamide (43), and cholesterol (46)—were found by GNPS spectral matching in MN_1_ (Table 4). MN_2_, composed of 42 nodes, matched the 7 known compounds 1, 8, 13, 44, 62, 68, and 69, and they were found to be amino acids and their derivatives (Table 4).

**Figure 6:**
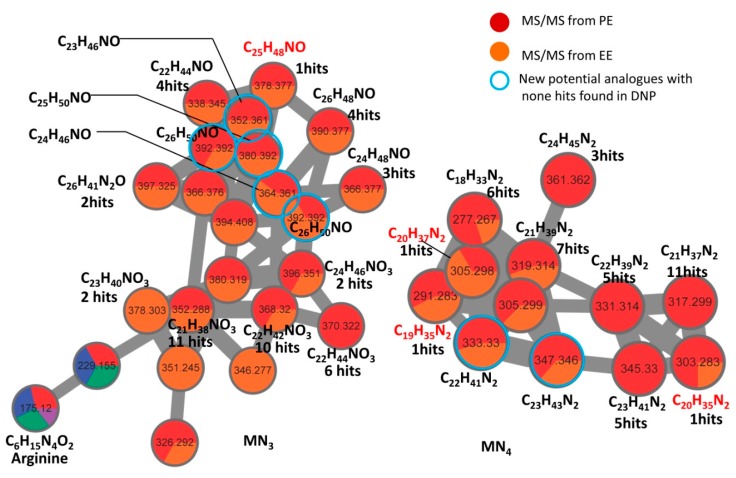
Manual annotation of molecular families MN_3,4_ by formula searching in online Dictionary of Natural Products (DNP, V27.2). The molecular formulae were predicted using "SmartFormula" function built in DataAnalysis software with *m*/*z* tolerance below 3 ppm. The predicted formulae represent [M + H]^+^ or [M + Na]^+^. Molecular formula in red indicates that there is only one hit matched in DNP, and new potential analogues are indicated with blue circles. The number of the hits matching to known formulae is presented under the predicted molecular formula.

However, the annotation rate of MN_1_ was 7.0%, which is lower than that of MN_2_. Therefore, a separate dereplication workflow *Dereplication* v1.2.5 (https://proteomics2.ucsd.edu/ProteoSAFe/index.jsp) was employed to decipher the chemical diversity. Applying this dereplication tool allows enhanced putative identification of known compounds in *Ligia* extracts with a cosine score cutoff value of 0.70. As listed in Table 4, 69 compounds covering all the GNPS matching hits were potentially identified by MS/MS spectral comparison. Of the five *Ligia* extracts, PE matched the largest number of hits with 46 compounds putatively identified, and EE ranked the second with 37 matching hits (Table S2). In MN_1_, 17 of the 43 nodes were annotated with known compounds and they were found to be fatty acids and fatty acid amides (Figure 5B). The annotation rate of MN_1_ was, therefore, greatly improved from 7.0% to 39.5%. However, we must point out that some nodes are labeled with two or three different hits in MN_1_. The phenomenon may result from applying the MS-cluster algorithm in generating molecular networks, which cannot distinguish between isomers with the same mass weight. According to the results, MN_2_, previously annotated as amino acids and their derivatives, was further validated with 6 more phenylalanine derivatives, 11, 14, 42, 48, 52, and 67, putatively identified by MS/MS spectral comparison (Figure 5B, Table 4). However, there were no hits matched in MN_3,4_. To reveal the chemical nature of the molecular families of MN_3,4_, manual dereplication was performed by searching the molecular formula in online Dictionary of Natural Products (DNP, V27.2). The results demonstrated that MN_3,4_ families were mainly composed of alkaloids with or without an oxygen atom (Figure 6). Importantly, some alkaloids bearing the molecular formula C_23_H_46_NO, C_24_H_46_NO, C_26_H_50_NO, C_23_H_43_N_2_, and C_22_H_41_N_2_ ([M + H]^+^), were strongly suggested as new undescribed analogues in *L. exotica*, since no hits were matched in the DNP by formula searching.

**Figure 7:**
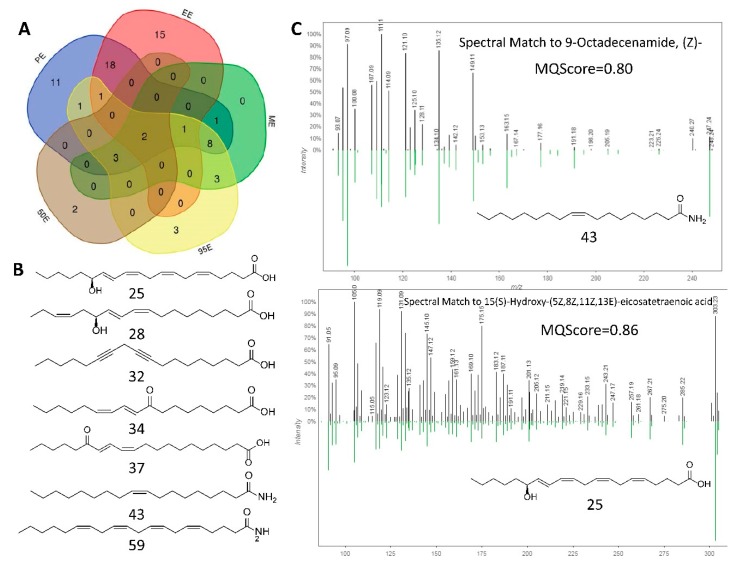
(**A**) The Venn diagram based on the dereplicated compounds 1–69 from PE, EE, ME, 95E, and 50E. (**B**) Representative structures of fatty acids and fatty acid amides identified with either PE or EE with a cosine score cutoff value of 0.70. (**C**) The representative matching results of two experimental MS/MS spectra matching to known compounds (*Z*)-9-octadecenamide (43) and 15(S)-hydroxy-(5Z,8Z,11Z,13E)-eicosatetraenoic acid (25). The green MS/MS spectra are from isolated reference compounds in the database. The similarity between the experimental MS/MS spectra and the reference MS/MS spectra is calculated as MQScore, which ranges from 0 to 1. The closer the MQScore is to 1, the greater the structural similarity.

To give a comprehensive view of the 69 putatively identified compounds in different *Ligia* extracts, a Venn diagram was drawn using a publicly available tool (Figure 7A, Figure S3). PE and EE were the best annotated extracts with 46 and 37 compounds identified by spectral matching, respectively. Moreover, PE and EE shared 18 hits, suggesting great similarity in chemical components. Besides, PE and EE also possessed the highest number of unique compounds that were not identified in ME, 95E, and 50E. Of the 18 compounds identified in both PE and EE, 9 hits were fatty acids with or without hydroxy (20, 22, 23, 24, 25, 26, 27, 28, 30, 31, 34). Figure 7B presents some representative structures of fatty acids and fatty acid amides dereplicated from either PE or EE. These lipid-like molecules were found to come from the same molecular cluster MN_1_, except for arachidonoyl amide (59). Figure 7C shows two experimental MS/MS results matching to known compounds (Z)-9-octadecenamide (43) and 15(S)-hydroxy-(5Z,8Z,11Z,13E)-eicosatetraenoic acid (25) with the MQScore values of 0.80 and 0.86, respectively. In addition, obvious features shared between the lipophilic PE and hydrophilic ME and 95E were six amino acid derivatives (3, 4, 8, 10, 11, 14) and two phosphatidylcholines (29, 40) out of 8 hits (Figure 7A, Figure S3).

## 3. Discussion

The sea slater *Ligia* spp. is currently used as a traditional Chinese medicine in southern Chinese coastal areas. The utilization of these marine species is based on a long tradition of use in folk medicine. However, the pharmaceutical value of the sea slater *Ligia* spp. was not included in Chinese Pharmacopoeia 2015 and its previous versions, which indicates that the biological effects of *Ligia* spp. still lack substantial scientific validation. Whether the sea slater *Ligia* spp. holds potential for development into new resources containing analgesic and anti-inflammatory compounds is of much interest. In the present study, we demonstrated, for the first time, the pronounced analgesic and anti-inflammatory effects of lipophilic extracts from the sea slater *L. exotica*. Deciphering the analgesic and anti-inflammatory PE with molecular networking revealed various lipophilic molecules, such as hydroxy fatty acids and fatty acid amides, which may contribute to understanding of the observed biological phenotypes and the pain-relief effects of *Ligia* spp. in folk medicine. More importantly, the present study detected two clusters of potentially new alkaloids, which highlights the potential for mining the chemical properties of *Ligia* spp.

In the present study, various extracts with different polarities were prepared from the hydroalcoholic extract (85%EtOH) of *L. exotica* by successive extraction procedure, which were demonstrated to contain different chemical profiles and yields. Importantly, differences were also observed in the following anti-inflammatory assays. PE and EE, instead of high polar ME, 95E, and 50E, were found to significantly inhibit the NO production in RAW264.7 macrophages. The phenomenon indicates that the anti-inflammatory compositions within *L. exotica* are mainly concentrated in the lipophilic fractions. NO is an important neurotransmitter, which could mediate the nociception process at both central and peripheral levels [21]. Therefore, this may represent an important finding to understand the pain-relief effects of *Ligia* spp. in folk medicine, considering the fact that sea slaters are usually immersed in Chinese spirits and the resulting liquor is used to relieve pain symptoms. Moreover, the anti-inflammatory PE was further demonstrated to have considerable analgesic effects in either the writhing test or hot plate test, which strongly indicates that PE may possess dual analgesic effects through peripheral and central mechanisms. Considering the chemical complexity of PE, the analgesic effects of PE may be the result of a combination of various chemicals acting together.

Abundant unsaturated fatty acids and their esters were identified in the biologically active PE and EE by GC-MS analysis (Figure 4 and Table 3). Ethyl esters—methyl esters of fatty acid—were the main esters detected in *L. exotica*. Of note, the extraction procedure using alcohol may result in the formation of fatty acid esters as artefacts. The fatty acid profiles of *L. exotica* featured high amounts of monounsaturated fatty acids (MUFAs), which was different with those of some common seafood organisms, such as the octopus *Octopus tetricus* and squid *Sepioteuthis australis* [22]. Substantial research has demonstrated the health-promoting effects of dietary monounsaturated fatty acids [23,24,25]. In this study, Z-9-hexadecenoic acid and 6-octadecenoic acid were detected as major compounds in the GC-MS chromatograms of PE and EE (Figure 4A and Table S1). The compound Z-9-hexadecenoic acid, also known as palmitoleic acid, was recently suggested as a novel anti-inflammatory mediator in murine models [26]. Moreover, as an isomer of Z-9-hexadecenoic acid, Z-7-hexadecenoic acid was found to be more potent than Z-9-hexadecenoic acid in anti-inflammatory activities in LPS-induced human monocytes or murine peritoneal macrophages [27]. The presence of Z-9-hexadecenoic acid may contribute to the anti-inflammatory effects of *L. exotica*.

The first global molecular map picturing the chemical phenotypes of *L. exotica* was illustrated in the present study by MS/MS-based molecular networking. The anti-inflammatory and analgesic PE was found to be the most chemically diverse part of the 85% EtOH extract, and it showed the highest similarity with EE in chemical compositions. The chemical similarity between PE and EE may explain their similar anti-inflammatory effects at 200 μg/mL. The fatty acids cluster MN_1_, together with the alkaloids clusters MN_3,4_, were distinguishing features of PE and EE, which differed from ME, 95E, and 50E in the molecular map of *Ligia* extracts. A considerable number of aliphatic compounds were detected in the MN_1_ cluster, including polyunsaturated fatty acids (26, 32) and their derivatives with hydroxy (23, 24, 25, 28, 35, 56), hydroperoxy (21), epoxides (20, 22, 57, 58) or keto (27, 34, 37) substituents, and fatty acid amides (43, 59). As the largest and best-annotated molecular family, MN_1_ represents an important chemotype to understand the anti-inflammatory and analgesic effects of PE. The 9,12-octadecadiynoic acid (32) identified in PE was able to inhibit cyclooxygenase and lipoxygenase at nanomolar concentrations [28,29]. In the present study, hydroxy fatty acids (23, 24, 25, 28, 35, 56) were putatively identified from fresh *L. exotica* with a cosine score cutoff value of 0.70 by spectral matching, of which 8-HETE (35) was also identified from its crustacean counterpart *Euphausia pacifica* [30]. The compound 13S-hydroxy-9Z,11E,15Z-octadecatrienoic acid (28) was reported to have potent anti-inflammatory activity by inactivating NLRP3 inflammasome complex through the peroxisome proliferator-activated receptor (PPARγ) pathway [31]. In addition, as a potent inhibitor against platelet 12-lipoxygenase (12-LO) and neutrophil 5-LO, 15(S)-hydroxy-(5Z,8Z,11Z,13E)-eicosatetraenoic acid (15-HETE, 25) was also demonstrated to possess anti-inflammatory effects [32].

Of note, a sub-cluster composed of (*Z*)-9-octadecenamide analogues was detected in MN_1_. Additionally, (Z)-9-octadecenamide (43) has been shown to exhibit various in vitro bioactivities, including direct inhibition of CB_1_ cannabinoid receptors, modulation of 5-HT receptors, and activation of TRPV1 vanilloid receptor [33]. Importantly, the analgesic effect of (*Z*)-9-octadecenamide was also demonstrated in the tail-flick test and hot-plate-test [34]. Besides (*Z*)-9-octadecenamide, arachidonoyl amide (59) was also detected within *L. exotica*. The compound arachidonoyl amide is an analog of anandamide (AEA), which is an effective analgesia acting through modulation of the peripheral CB_1_ receptor [35]. The arachidonoyl amide had a similar effect but its binding activity to CB_1_ receptor was weaker than AEA [36,37]. In addition, the second largest molecular family MN_2_, mainly composed of phenylalanine derivatives, was not considered to contribute to the anti-inflammatory and analgesic effects of *L. exotica* due to their predominant presence in ME, 95E, and 50E.

In conclusion, we demonstrated, for the first time, the ethnopharmacological value of the sea slater *L. exotica* as a promising source of anti-inflammatory and analgesic compounds. The observed anti-inflammatory and analgesic effects of *L. exotica* were largely attributed to the lipophilic extracts PE and EE. As indicated by GC-MS, aliphatic molecules, such as unsaturated fatty acids and their esters, dominated in the volatile components of PE and EE. The global chemical phenotypes within *L. exotica* were found to be diverse, and as many as 23 molecular families were revealed by applying MS/MS-based molecular networking. Besides the well annotated fatty acids cluster and phenylalanine derivatives cluster, another two molecular families composed of unknown alkaloids were tentatively identified, in which novel alkaloids were strongly suggested. Moreover, the biologically active PE and EE were found to be mainly composed of fatty acids and alkaloids. Literature investigations of the putatively identified compounds strongly suggested monohydroxylated fatty acids and fatty acid amides as important effector molecules within *L. exotica*. The biological roles of alkaloids clusters MN_3,4_ were not determined in this study. Considering the potentially structural novelty within these alkaloids, further isolation and identification of these novel alkaloids from sea slaters should be addressed.

## 4. Materials and Methods

### 4.1. Sample Collecting

*L. exotica* species were collected from Zhoushan Islands (30°00′58.7″ N 122°20′33.1″ E), Zhejiang Province, China, in the summer of 2017. A voucher specimen was deposited in the Key Laboratory of Experimental Marine Biology, Institute of Oceanology, Chinese Academy of Sciences.

### 4.2. Successive Extraction

Freshly collected *L. exotica* (wet weights, 6.54 kg) were immediately shipped to the laboratory and extracted four times with 85% ethanol (25 L for each extraction) for a month. After filtration, the ethanol extracts were combined and concentrated under reduced pressure to afford a crude 85% ethanol extract (85% EtOH) with a unique smell. The crude extract (approximate 1 L in volume) was then extracted three times with petroleum ether (3 L), and the combined petroleum ether extract was concentrated under reduced pressure to yield a petroleum ether extract (PE, 76.9 g). Then, the residual crude extract was further extracted in a similar way with ethyl acetate, methanol, 95% ethanol, and finally 50% ethanol, respectively, to obtain ethyl acetate extract (EE, 8.0 g), methanol extract (ME), 95% ethanol extract (95E, 22.2 g), and 50% ethanol extract (50E, 80.9 g). No residue was insoluble after the above-mentioned successive extraction procedures. Of note, ME was concentrated under reduced pressure to afford insoluble material ME-3 (61.3 g). Then, the methanol-soluble supernatant was subject to alcohol precipitation with 95% ethanol to afford the sediments ME-2 (180.1 g) and the 95%-ethanol-soluble ME-1 (111.4 g).

### 4.3. High-Performance Liquid Chromatography (HPLC) with Diode Array Detector

Twenty microliters of the above mentioned *Ligia* extracts, PE, EE, ME-1, ME-2, ME-3, 95E, and 50E, were analyzed with an Agilent HPLC device coupled to an diode array detector and a C18 reversed phase column (Waters, Xbridge, 250 × 4.6 mm, 5 μm) was used. The HPLC profiling was performed at room temperature, and the flow rate was set at 1 mL/min. The mobile phases consisted of purified water (A) and methanol (B). The elution gradient was set as follows: 0–5 min, 5% B; 5–40 min, 5-100% B; 40–55 min, 100% B; 55–60 min, 100–5% B.

### 4.4. Anti-Inflammatory Activity 

The anti-inflammatory activities of *Ligia* extracts were determined by measuring nitric oxide (NO) content in LPS-induced RAW264.7 macrophages [38]. The RAW264.7 macrophages were purchased from Stem Cell Bank, Chinese Academy of Sciences (Shanghai, China) and cultivated at 37 °C in 10% Fetal Bovine Serum (FBS) supplemented Dulbecco’s Modified Eagle Medium (DMEM) in a humidified atmosphere of 5% CO_2_. The RAW 264.7 macrophage cells in 10% FBS DMEM were further incubated in 96-well plates (1 × 10^5^ cells/well) for 12 h. Then, the cells were stimulated with LPS (1 μg/mL), while at the same time *Ligia* extracts (PE, EE, ME-1, ME-2, ME-3, 95E, final concentrations, 200 μg/mL) were added into the media and incubated for 20 h at 37 °C. After incubation, NO production was determined by measuring the nitrite concentration in the supernatant using Griess reagent (Sigma, St. Louis, MO, USA). Briefly, 100 μL of the supernatant were translated to a new 96-well plate and an equal volume of Griess reagent was added and gently mixed. After incubation at room temperature for 10 min, the absorbance at 570 nm was measured using an Infinite M100 plate reader (Tecan Group Ltd., Männedorf, Switzerland). *N*-monomethyl-_L_-arginine (L-NMMA, Sigma, St. Louis, MO, USA) and dimethyl sulfoxide (DMSO) were used as positive and negative controls, respectively. The IC_50_ value (50% concentration of inhibition) of PE was calculated using the Reed and Muench method [39]. All experiments were performed in three independent replicates.

### 4.5. In Vivo Test

Institute of Cancer Research (ICR) mice were used in this study, and they were obtained from Qingdao Pharmaceutical Inspection Institute. The ICR mice were kept in stainless steel cages for one week in laboratory conditions before experimental incubation. All experimental procedures were approved by the Committee on the Ethics of Animal Experiments of the Institute of Oceanology, Chinese Academy of Sciences. Animals were kept in animal care facility under controlled temperature, humidity, and light/dark cycle, and had access to food and water ad libitum. Efforts were made to minimize the number of animals used in the study and unnecessary suffering during the experimental process, according to Guide for the Care and Use of Laboratory Animals [40].

#### 4.5.1. Writhing Test

The acetic-acid-induced writhing test was performed according to a previous described method [41] with minor modifications. Male and female ICR mice were randomly grouped into 7 groups of 6–8 ICR mice, and they were transferred to the lab to adapt to the surroundings before the experiments. The mice (*n* = 6–8) were subject to intragastric administration of 0.5% sodium carboxymethylcellulose solution (CMC-Na, negative control), 20 mg/kg indometacin (positive control), or *Ligia* extracts (PE, 200 or 600 mg/kg; ME-1, 600 mg/kg; 95E, 600 mg/kg; 50E, 600 mg/kg) in a final volume of 1 mL. One hour later, the mice were intraperitoneally (i.p) injected with 0.8% acetic acid at a dose of 10 mL/kg. Then, the animals were immediately placed into new cages, which were separated into four individual spaces, to observe the mice’s behavior. A complete acetic-acid-evoked writhing consists of abdominal constrictions coupled with a stretching of at least one hind limb. The number of the abdominal constrictions, which was recorded as an index of analgesic potency of assayed samples compared to the control group, was counted within 25 min.

#### 4.5.2. Hot Plate Test

The hot-plate test was used to measure the central analgesic effect of PE according to the previously described method [42]. The assay was performed with the YLS-6B hotplate analgesia meter (Beijing Zhongshidichuang Co., Beijing, China). The temperature was set at 55 ± 0.2 °C. The latency time (s) of occurrence of pain-like behaviors, such as licking paws or jumping, was recorded after incubation of the experimental animals on the hot plate. The female mice with response latency times below 15 s were selected 2 h prior to the test. The selected animals were randomly grouped and placed in the lab to adapt to the experimental environments. Then, the mice (*n* = 10) were pre-treated with a 0.5% CMC-Na solution (negative control), 20 mg/kg tramadol (positive control), or PE (200 or 600 mg/kg), respectively. 1 h later, the mice were placed on the metal surface of the hotplate with a cutoff time of 30 s. The latency times (s) were recorded at 0, 0.5 h, 1 h, 2 h, and 48 h after intragastric administration of sample solutions.

### 4.6. Antioxidant Activity

The antioxidant activities of *Ligia* extracts were determined using 2,2′-azino-bis(3-ethylbenzthiazoline-6-sulfonic acid (ABTS) as the color developing agent. The assays were performed with the Total Antioxidant Capacity Assay Kit (Product code, S0119, Beyotime Institute of Biotechnology, Shanghai, China), according to the product’s instructions. Briefly, a stock solution was prepared by mixing the ABTS solution (S0119-1) and potassium persulphate (S0119-2) at a volume ratio of 1:1. Then, the ABTS+ working solution was prepared by diluting the stock solution with 80% ethanol to an absorbance of 0.70 ± 0.05 at 734 nm using Infinite M100 plate reader (Tecan Group Ltd., Männedorf, Switzerland). PE and EE were diluted in 80% ethanol and other *Ligia* extracts were diluted in sodium phosphate buffer (pH 7.0). Then, 10 μL of the diluted samples (final concentrations, 100 μg/mL) were added into 200 μL ABTS+ working solution, and the absorbance of the reaction mixtures was measured at 734 nm after 6 min of incubation at room temperature. Trolox (final concentration, 95 μg/mL) was used as positive control. The relative activity was calculated by using a Trolox calibration curve. The concentration–response curve of 50E was fitted with a four-parameter logistic curve in GraphPad Prism 6.0 and the IC_50_ was calculated in the software (GraphPad software, San Diego, CA, USA).

### 4.7. Gas Chromatography-Mass Spectroscopy (GC-MS)

GC-MS analysis of PE and EE was performed using an Agilent 7890A/5975C GC-MS system equipped with a capillary column HP-5MS (30 m × 0.250 mm, 0.25 micron, Agilent Technologies, Inc., Santa Clara, CA, USA). Before the experiments, PE was pretreated by silica gel chromatography to remove polar components. The silica gel vacuum liquid chromatography was performed using a petroleum ether-ethyl acetate (0:100 - 100:0) and dichloromethane-methanol-water (10:1:0 - 1:2:0 - 0:0:1) solvent system. Fractions were combined according to their HPLC profiles, and five fractions of Fr.1-5 were finally obtained (Figure S2). The PE fraction Fr.5 was completely water-soluble and was omitted from the GC-MS analysis. A milligram aliquot of Fr.1-4 and EE was employed in GC-MS analysis and the split ratio was set at 10:1. The experimental parameters were set as follows: ionizing energy, 70 eV; Helium gas (99.999%) was used as the carrier gas, with a flow rate of 1 mL/min; injector temperature, 250 °C; on-source temperature, 280 °C; total running time, 35.0 min. A blank solvent control (methanol) was also subject to the GC-MS analysis under the same conditions to remove the possible contaminants. To identify compounds, the database of National Institute Standard and technology (NIST) was employed. The spectrum of the unknown component in PE and EE was compared with the spectrum of the known components stored in the NIST library, and the similarity between two spectrum was expressed as matching degree (%). Compounds with a matching degree value above 95% and shown only in the *Ligia* extracts were interpreted as valid hits. The relative % amount of each component was calculated by comparing its average peak area to the total areas.

### 4.8. MS/MS-Based Molecular Networking and Dereplication

Five microliters of *Ligia* extracts (3.0–4.6 mg/mL) or solvent control (methanol) were subject to HPLC-MS/MS profiling using a Agilent HPLC system coupled to a maxis micrOTOF-Q plus mass spectrometer (Bruker Daltonics, Boston, MA, USA) equipped with an electrospray ionization (ESI) source. A Kinetex C18 reversed phase column (50 × 2.1 mm) (Phenomenex, Torrance, CA, USA) was employed and the isolation was performed with a flow rate of 0.35 mL/min. The column was equilibrated with 10% phase B (acetonitrile with 0.1% formic acid; phase A, water with 0.1% formic acid), and the chromatographic conditions were set as follows: 0–3 min, 10% B; 3–23 min, 10–100% B; 23–30 min, 100% B; 30–35 min, 100–10% B. The MS data were acquired in positive mode using an MS range of *m*/*z* 50–1500. An external calibration with sodium formate (Agilent technologies Inc., Santa Clara, CA, USA) was conducted prior to each data collection throughout the runs. The MS parameters were set as follows: nebulizer gas pressure, 4.5 Bar; dry gas flow, 9 L/min; capillary voltage, 3500 V; ion source temperature, 220 °C. Auto MS/MS fragmentation was carried out for the five most intense ions per spectrum. A gradient of collision-induced dissociation (CID) energy from 20 to 50 eV was used according to the parent mass from 100 Da to 2000 Da. A Bruker Compass DataAnalysis 4.2 software (Bruker Daltonics, Boston, MA, USA) was used to check and analyze the MS data.

The raw MS/MS data were converted to mzXML files using the DataAnalysis 4.2 software, and the files were submitted to the Global Natural Products Social (GNPS) Molecular Networking web-platform (http://gnps.ucsd.edu) for creating molecular networks [43]. Briefly, the data were filtered with default parameters, and then clustered with the activated MS-Cluster. The parent ion mass tolerance was set to 0.05 Da, and the MS/MS fragment ion mass tolerance of 0.02 Da was used. Concensus spectra that contained less than 2 spectra were removed from the MS/MS data. Networks were generated using a cosine score above 0.70 and 6 minimum matched peaks. The MS/MS spectra in the network were searched against the GNPS spectral libraries. For visualization and more specific analysis, the network data were exported into Cytoscape (Version 3.6, Cytoscape consortium, San Diego, CA, USA) for visualization.

Further dereplication of known compounds in *Ligia* extracts was conducted with a separate workflow *Dereplication* v1.2.5 (https://proteomics2.ucsd.edu/ProteoSAFe/index.jsp, available until 2019-04-25). The dereplication parameters were given as follows: precursor ion mass tolerance, 0.02 Da; fragment ion tolerance, 0.01 Da. Only compounds that shared at least 6 matched peaks and had a cosine score above 0.70 were considered to be hits. All the potential hits identified by spectral matching in PE, EE, ME, 50E, and 95E were exported into an Excel file and subject to compound statistics, which were further uploaded to an online tool (http://bioinformatics.psb.ugent.be/webtools/Venn/, available until 2019-04-25) for generating the venn diagram.

### 4.9. Statistical Analysis 

The data are expressed as the means ± S.E.M of three independent experiments. One-way analysis of variance (ANOVA) test was used for statistical analysis, followed by a Tukey’s post hoc test for multiple comparisons built in Graphpad Prism 6.0 (GraphPad Software Inc., San Diego, CA, USA); * *p* < 0.05, ** *p* < 0.01, *** *p* < 0.001 are considered statistically significant.

## Figures and Tables

**Table 1:** The yield of *Ligia* extracts from fresh *L. exotica* biomass.

Extracts ^a^	85% EtOH	PE	EE	ME-1	ME-2	ME-3	95E	50E
Weight(g)	540.8	76.9	8.0	111.4	180.1	61.3	22.2	80.9
%(total weight ^b^)	100	14.2	1.5	20.6	33.3	11.3	4.1	14.9
%(wet weight ^c^)	8.3	1.2	0.1	1.7	2.7	0.9	0.3	1.2

Note: different organic solvents, petroleum ether, ethyl acetate, methanol, and EtOH were used in the successive extraction process to obtain extracts with different polarity, the percentage of single *Ligia* extract to the weight of the 85% EtOH extract, and the percentage of single *Ligia* extract to the wet weight of *L. exotica*.

**Table 2:** The analgesic effects of PE in hot-plate test assayed with Institute of Cancer Research (ICR) mice. The antinociceptive activity of PE was expressed as latency time (s), which indicates the heat tolerance of the experimental animals.

Groups	Latency Time (s)				
	0 h	0.5 h	1 h	2 h	48 h
BLANK	11.55 ± 0.61	11.87 ± 0.85	11.23 ± 0.75	12.07 ± 0.75	—
PE (200 mg/kg)	12.37 ± 0.62	14.10 ± 0.65	16.98 ± 0.69 ***	16.91 ± 1.10 ***	12.49 ± 0.64
PE (600 mg/kg)	16.67 ± 1.19	27.64 ± 2.15 ^###^	24.06 ± 1.56 ^#^	28.61 ± 2.41 ^###^	17.69 ± 1.78
Tramadol (20 mg/kg)	12.50 ± 0.48	19.29 ± 1.21 ^§§§^	19.02 ± 1.42 ^§§§^	19.30 ± 1.28 ^§§§^	—

**Notes:** Results are expressed as mean ± standard error of the mean (S.E.M, *n* = 8–10); ^#^ *p* < 0.01, *** *p*, ^###^ *p*, ^§§§^ *p* < 0.001 are considered significantly different to their respective control group at 0 h.

**Table 3:** Putative identification of compounds within PE and EE extracts from *L. exotica* by GC-MS with a matching degree cutoff value of 95%.

Peak Number	RT ^a^ (min)	Compound_Name	Chemical Formula	*m*/*z*^b^ (Da)	MD ^c^ (%)	PE F1 ^d^	PE F2 ^d^	PE^c^ F3 ^d^	PE F4 ^d^	EE
1	12.056	1-pentadecene	C_15_H_30_	210	99	+				
2	13.089	2(4H)-benzofuranone, 5,6,7,7a-tetrahydro-4,4,7a-trimethyl-	C_11_H_16_O_2_	180	96		+			
3	16.388	8-heptadecene	C_17_H_34_	238	95	+				
4	18.029	Tetradecanoic acid	C_14_H_28_O_2_	228	95		+			
5	18.67	Tetradecanoic acid, ethyl ester	C_16_H_32_O_2_	256	97	+	+			
6	21.762	Hexadecenoic acid, Z-11-	C_16_H_30_O_2_	254	99	+				
7	21.927	Z-9-hexadecenoic acid	C_16_H_30_O_2_	254	99		+	+		+
8	22.160	n-hexadecanoic acid	C_16_H_32_O_2_	256	99		+	+		
9	22.207	Ethyl 9-hexadecenoate	C_18_H_34_O_2_	282	99	+	+	+		
10	22.604	Hexadecanoic acid, ethyl ester	C_18_H_36_O_2_	284	97	+	+			
11	23.526	Z-10-heptadecenoic acid	C_17_H_32_O_2_	268	97		+			
12	24.458	10,13-octadecadienoic acid, methyl ester	C_19_H_34_O_2_	294	99			+		
13	24.564	9-octadecenoic acid, methyl ester, (E)-	C_19_H_36_O_2_	296	99			+		
14	24.675	cis-13-octadecenoic acid, methyl ester	C_19_H_36_O_2_	296	99			+		
15	25.204	9,12-octadecadienoic acid (Z,Z)-	C_18_H_32_O_2_	280	99	+	+	+		+
16	25.331	E-13-octadecenoic acid	C_18_H_34_O_2_	282	99	+				
17	25.390	6-Octadecenoic acid	C_18_H_34_O_2_	282	99	+	+	+		+
17	25.432	6-Octadecenoic acid, (Z)-	C_18_H_34_O_2_	282	99			+		
18	25.566	9,12-octadecadienoic acid, ethyl ester	C_20_H_36_O_2_	308	99	+				
19	25.660	9,17-octadecadienal, (Z)-	C_18_H_32_O	264	96			+		
20	25.665	Linoleic acid ethyl ester	C_20_H_36_O_2_	308	99		+			
21	25.766	Ethyl oleate	C_20_H_38_O_2_	310	99	+	+			
22	28.89	5,8,11,14-eicosatetraenoic acid, ethyl ester, (all-Z)-	C_22_H_36_O_2_	332	95	+	+			
23	29.060	9,12,15-octadecatrien-1-ol, (Z,Z,Z)-	C_18_H_32_O	264	95		+			
24	39.332	Cholesterol	C_27_H_46_O	386	99	+	+			
Sum up						13	15	10	0	3

Note: RT = retention time (min); *m*/*z* = mass-to-charge ratio; MD = Matching Degree, which indicates the structural similarity. The values of MD range from 0 to 100%; PE-F1 = PE-Fr.1; PE-F2 = PE-Fr.2; PE-F3 = PE-Fr.3; PE-F4 = PE-Fr.4.

**Table 4:** Compounds putatively identified from all the five *Ligia* extracts using *Dereplication* v1.2.5.

Comps. ^a^	RT ^b^ (s)	Precursor MZ ^c^ (Da)	Compound_Name	Shared Peaks ^d^	MQScore ^e^
1	70.802	166.087	Phenylalanine	6	0.956563
	70.802	166.083	DL-phenylalanine	17	0.926237
2	82.189	205.098	Tryptophan	25	0.954918
	82.189	205.097	l-tryptophan	30	0.952962
3	83.523	188.07	Abrine	10	0.920207
4	83.523	188.071	DL-indole-3-lactic acid	13	0.921548
5	96.254	265.154	Phe-Val	19	0.787928
6	127.29	231.114	1,2,3,4-tetrahydroharmane-3-carboxylic acid	10	0.85109
7	147.378	136.076	DL-octopamine	6	0.728619
8	151.331	279.17	Spectral Match to Phe-Leu from NIST14	14	0.923904
9	171.655	279.169	Spectral Match to Leu-Phe from NIST14	7	0.718983
10	214.147	279.169	Spectral Match to Phe-Ile from NIST14	8	0.909698
11	260.648	208.097	N-acetylphenylalanine	12	0.836929
	260.648	208.097	l-phenylalanine, *N*-acetyl- from NIST14	10	0.91052
12	287.152	245.128	Pyrrolo[1,2-a]pyrazine-1,4-dione, hexahydro-3-(phenylmethyl)-	18	0.718626
13	287.152	245.128	Phenylalanine, prolyl-	19	0.747031
14	292.27	313.155	Phe-Phe from NIST14	9	0.95749
15	393.38	164.107	*N*-acetyl-2-phenylethylamine	7	0.8942
16	883.698	333.206	5-[2-(3-Furyl)ethyl]-8-hydroxy-5,6,8a-trimethyl-3,4,4a,5,6,7,8,8a-octahydro-1-naphthalenecarboxylic acid	168	0.727051
17	883.698	333.206	5-[2-(3-Furyl)ethyl]-8a-(hydroxymethyl)-5,6-dimethyl-3,4,4a,5,6,7,8,8a-octahydro-1-naphthalenecarboxylic acid	169	0.730714
18	1018.02	415.211	2H-oxireno[1,10a]phenanthro[3,2-b]furan-10(11bH)-one, 5,7-bis(acetyloxy)-3,3a,4,5,6,7,7a,7b,8,8a-decahydro-4,4,7a,11-tetramethyl-, (1aS,3aR,5S,7S,7aR,7bS,8aR,11bR)-	49	0.887886
19	1018.02	415.211	6-[3-[(3,4-dimethoxyphenyl)methyl]-4-methoxy-2-(methoxymethyl)butyl]-4-methoxy-1,3-benzodioxole	25	0.896878
20	1101.95	301.215	Spectral Match to 14(15)-EpETE from NIST14	147	0.808564
21	1135.87	293.211	Spectral Match to 9(S)-HpOTrE from NIST14	52	0.712923
22	1148.93	301.216	Spectral Match to 17(18)-EpETE from NIST14	127	0.809739
23	1148.93	301.216	(.+/-.)-8-Hydroxy-5Z,9E,11Z,14Z,17Z-eicosapentaenoic acid from NIST14	134	0.814645
24	1170.15	303.231	11S-hydroxy-5Z,8Z,12E,14Z-eicosatetraenoic acid	150	0.852725
25	1170.15	303.231	15(S)-hydroxy-(5Z,8Z,11Z,13E)-eicosatetraenoic acid from NIST14	150	0.865231
26	1172.17	279.231	Spectral Match to Pinolenic acid from NIST14	96	0.773502
27	1176.45	295.226	13-keto-9Z,11E-octadecadienoic acid from NIST14	86	0.820771
28	1178.25	277.216	13S-hydroxy-9Z,11E,15Z-octadecatrienoic acid	68	0.76488
29	1188.09	482.36	1-hexadecyl-sn-glycero-3-phosphocholine	7	0.887771
30	1191.89	317.211	9-hydroxy-1,4a-dimethyl-7-propan-2-yl-2,3,4,9,10, 10a-hexahydrophenanthrene-1-carboxylic acid	114	0.732695
31	1191.89	317.211	12-oxopimara-9(11),15-dien-18-oic acid	132	0.727094
32	1200.88	277.216	9,12-octadecadiynoic acid from NIST14	63	0.707527
33	1211.54	317.211	7-ethenyl-1,4a,7-trimethyl-6-oxo-2,3,4,8,8a,9,10,10a-octahydrophenanthrene-1-carboxylic acid	51	0.713914
34	1216.52	295.227	9-oxo-10E,12Z-octadecadienoic acid from NIST14	97	0.798115
35	1222.44	303.232	8S-hydroxy-5Z,9E,11Z,14Z-eicosatetraenoic acid	90	0.75798
36	1227.3	279.159	Spectral Match to Dibutyl phthalate from NIST14	9	0.923685
37	1290.23	323.258	Spectral Match to Eicosanoids_15-oxoEDE	45	0.722368
	1290.23	323.258	Spectral Match to 15-OxoEDE from NIST14	54	0.727381
38	1293.26	323.258	1-Naphthalenecarboxylic acid, decahydro-5-(5-hydroxy-3-methylpentyl)-1,4a-dimethyl-6-methylene-, (1R,4aS,5R,8aS)-	107	0.736152
39	1316.52	552.401	1-arachidoyl-2-hydroxy-sn-glycero-3-phosphocholine from NIST14	15	0.854794
40	1318.46	510.391	Spectral Match to Lyso-PAF C-18 from NIST14	9	0.893974
35	1349.1	303.23	Spectral Match to 8-HETE from NIST14	56	0.746991
41	1370.87	307.263	Spectral Match to Linolenic acid ethyl ester	64	0.809892
42	1389.49	402.301	(Z)-N-hexadec-9-enoyl-L-phenylalanine	17	0.878151
43	1408.8	282.279	Spectral Match to 9-octadecenamide, (Z)-	37	0.795687
44	1463.23	404.316	2-(14-methylpentadecanoylamino)-3-phenylpropanoic acid	26	0.90229
45	1866.48	369.351	Cholestan-3-one, (5.alpha.)- from NIST14	27	0.75334
46	1866.48	369.352	Spectral Match to Cholesterol from NIST14	24	0.763294
47	89.587	261.123	Pyrrolo[1,2-a]pyrazine-1,4-dione, hexahydro-3-[(4-hydroxyphenyl)methyl]-	53	0.734621
48	115.327	180.102	*N*-acetyltyramine	9	0.811762
49	334.623	197.117	Loliolide	38	0.765238
	334.623	197.117	2(4H)-Benzofuranone, 5,6,7,7a-tetrahydro-6-hydroxy-4,4,7a-trimethyl-, (6S,7aR)-	60	0.790237
50	354.718	146.06	Spectral Match to 1H-indole-4-carboxaldehyde from NIST14	6	0.84395
51	356.703	284.139	cyclo(D-Trp-L-Pro)	16	0.950215
	356.703	284.139	3-(1H-indol-3-ylmethyl)-2,3,6,7,8,8a-hexahydropyrrolo[1,2-a]pyrazine-1,4-dione	17	0.933498
52	629.326	261.159	cyclo(Phe-Leu)	34	0.737895
53	662.681	334.155	3-benzyl-6-(1H-indol-3-ylmethyl)piperazine-2,5-dione	13	0.888409
54	684.967	295.129	Aspartame|3-amino-4-[(1-benzyl-2-keto-2-methoxy-ethyl)amino]-4-keto-butyric acid	11	0.751393
	684.967	295.129	Aspartame|3-amino-4-[(1-benzyl-2-keto-2-methoxy-ethyl)amino]-4-keto-butyric acid	14	0.750524
55	970.585	321.242	5-(1,2,4a,5-tetramethyl-7-oxo-3,4,8,8a-tetrahydro-2H-naphthalen-1-yl)-3-methylpentanoic acid	143	0.800957
	970.585	321.242	5-[(1S,2R,4aR)-1,2,4a,5-tetramethyl-7-oxo-3,4,8,8a-tetrahydro-2H-naphthalen-1-yl]-3-methylpentanoic acid	152	0.804541
56	1187.73	327.231	(.+/-.)-11-hydroxy-4Z,7Z,9E,13Z,16Z,19Z-docosahexaenoic acid from NIST14	82	0.733423
57	1187.73	327.23	Spectral Match to 19(20)-EpDPE from NIST14	80	0.725253
58	1261.82	297.242	Spectral Match to 9(10)-EpOME from NIST14	36	0.702697
59	1322.28	304.26	Spectral Match to Arachidonoyl amide	74	0.857892
60	1471.59	358.31	Spectral Match to phenylethylamide 357	46	0.81775
61	1551.27	628.187	2-[3,4-bis[[(2S,3R,4S,5S,6R)-3,4,5-trihydroxy-6-(hydroxymethyl)oxan-2-yl]oxy]phenyl]-5,7-dihydroxychromen-4-one	28	0.741822
62	132.068	263.138	Spectral Match to Phe-Pro from NIST14	14	0.772827
63	386.204	352.165	Spectral Match to Phe-Trp from NIST14	9	0.880003
64	506.815	352.165	Spectral Match to Trp-Phe from NIST14	9	0.815122
65	75.058	229.16	Spectral Match to Leu-Pro from NIST14	6	0.792858
66	200.695	332.218	Spectral Match to Thr-Val-Leu from NIST14	7	0.732416
67	207.742	277.119	Spectral Match to PyroGlu-Phe from NIST14	16	0.73009
68	62.857	182.081	Spectral Match to L-Tyrosine from NIST14	11	0.947038
69	99.71	295.128	Spectral Match to Glu Phe from METLIN	9	0.777446

Note: Comps. = compounds potentially identified by MS/MS spectral comparison; RT = retention time (s); precursor MZ indicates mass weights (Da) of the precursors for detecting MS/MS fragments; shared peaks indicate the number of MS/MS fragments shared between the experimental spectra and the reference spectra; MQScore suggests the chemical similarity and the MQScore value ranges from 0 to 1.

## Supplementary Materials

The following are available online at https://www.mdpi.com/1660-3397/17/7/395/s1. Figure S1: UV absorbance of peaks occurred on the HPLC chromatograms of PE (A) and EE (B) from 200 nm to 450 nm. Figure S2: HPLC chromatograms of the combined PE fractions Fr.1-5 (A-E) at 220 nm. Figure S3: Outputs of venn diagram using the compounds 1-69 identified from PE, EE, ME, 95E, 50E by MS/MS-based spectral matching. The compound names have been shown in Table 4, and visualization of the results is presented in Figure 7A. Table S1: The relative content of the major compounds identified from PE and EE by comparing their respective peak area to the total areas. Table S2: Compounds putatively identified from *Logia* extracts by MS/MS spectral comparison with cosine score value of 0.70.
