# Supplementary material for: Integrated Gas Chromatograph-Mass Spectrometry (GC/MS) and MS/MS-Based Molecular Networking Reveals the Analgesic and Anti-Inflammatory Phenotypes of the Sea Slater Ligia exotica
Source: Mar Drugs. 2019 Jul 4;17(7):395. doi: 10.3390/md17070395 (PMC6669569; doi:10.3390/md17070395)
Supplement: Supplementary file 1 [file marinedrugs-17-00395-s001.pdf]

Supplementary data

Integrated Gas Chromatograph-Mass Spectrometry (GC/MS) and MS/MS-Based Molecular Networking Reveals the Analgesic and Anti-Inflammatory Phenotypes of the Sea Slater *Ligia exotica*

Yang Yue [1,2,3], Quanbin Zhang [1,2,3,*] and Jing Wang [1,2,3]

[1] Key Laboratory of Experimental Marine Biology, Institute of Oceanology, Chinese Academy of Sciences, 7 Nanhai Road, Qingdao 266071, China; yueyang@qdio.ac.cn(Y.Y.), qbzhang@qdio.ac.cn(Q.Z.); jingwang@qdio.ac.cn(J.W.)

[2] Laboratory for Marine Biology and Biotechnology, Qingdao National Laboratory for Marine Science and Technology, Qingdao, 266237, China; yueyang@qdio.ac.cn(Y.Y.), qbzhang@qdio.ac.cn(Q.Z.); jingwang@qdio.ac.cn(J.W.)

[3] Center for Ocean Mega-Science, Chinese Academy of Sciences, 7 Nanhai Road, Qingdao, 266071, China; yueyang@qdio.ac.cn(Y.Y.), qbzhang@qdio.ac.cn(Q.Z.); jingwang@qdio.ac.cn(J.W.)

* Correspondence: qbzhang@qdio.ac.cn(Q.Z.); Tel.: +86-0532-82898703

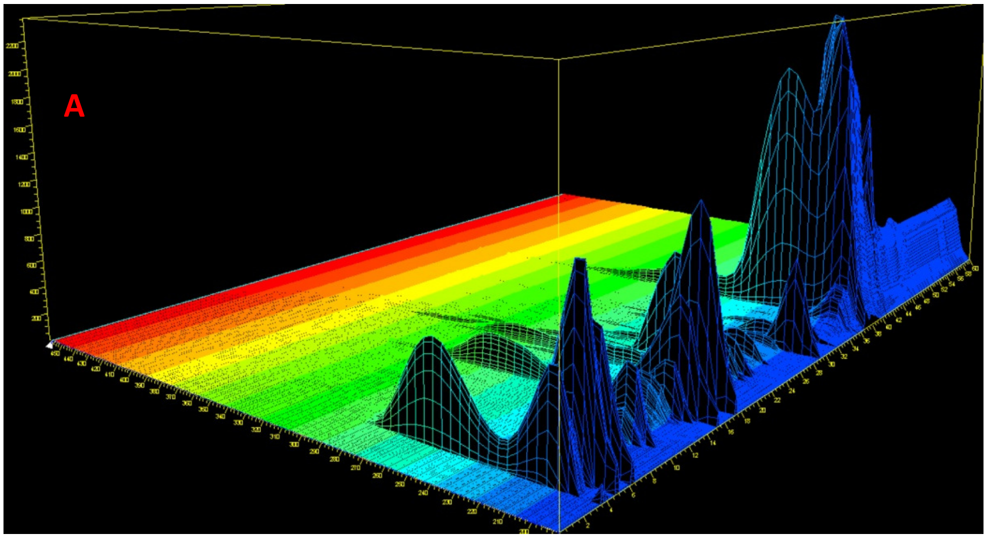

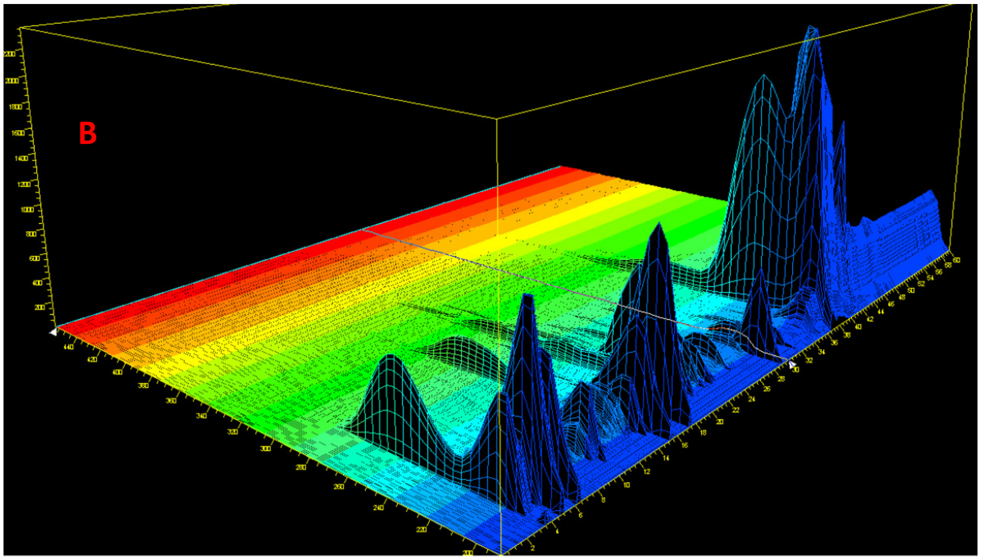

Figure S1. UV absorbance of peaks occurred in the HPLC chromatograms of PE (A) and EE (B) from 200 nm to 450 nm.

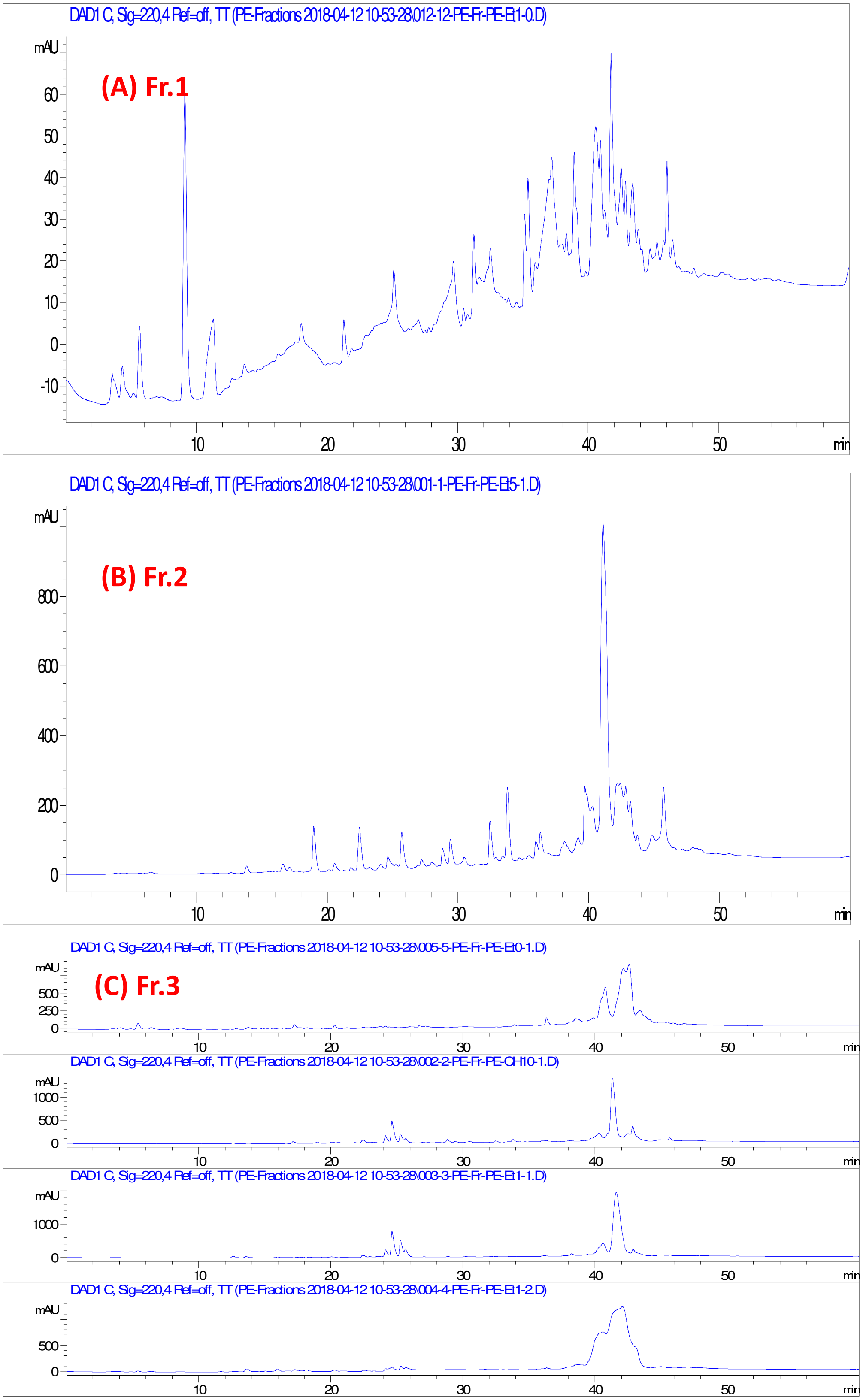
DAD1 C, Sig=220,4 Ref=off, TT (PE-Fractions 2018-04-12 10-53-28\012-12-PE-Fr-PE-Et1-0.D)
(A) Fr.1
mAU
min
DAD1 C, Sig=220,4 Ref=off, TT (PE-Fractions 2018-04-12 10-53-28\001-1-PE-Fr-PE-Et5-1.D)
(B) Fr.2
mAU
min
DAD1 C, Sig=220,4 Ref=off, TT (PE-Fractions 2018-04-12 10-53-28\005-5-PE-Fr-PE-Et0-1.D)
(C) Fr.3
mAU
min
DAD1 C, Sig=220,4 Ref=off, TT (PE-Fractions 2018-04-12 10-53-28\002-2-PE-Fr-PE-CH10-1.D)
mAU
min
DAD1 C, Sig=220,4 Ref=off, TT (PE-Fractions 2018-04-12 10-53-28\003-3-PE-Fr-PE-Et1-1.D)
mAU
min
DAD1 C, Sig=220,4 Ref=off, TT (PE-Fractions 2018-04-12 10-53-28\004-4-PE-Fr-PE-Et1-2.D)
mAU
min

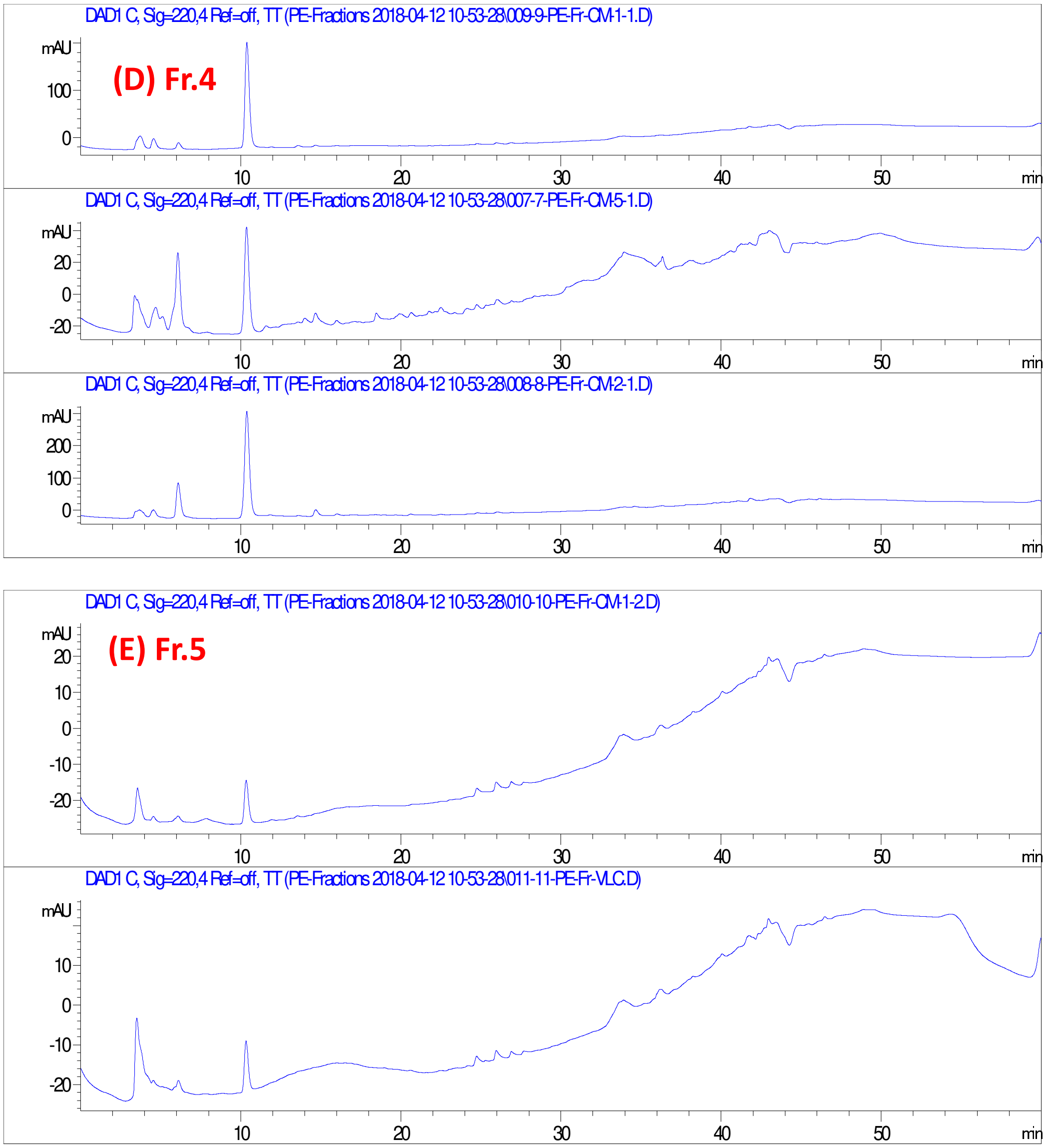

Figure S2. HPLC chromatograms of the combined PE fractions Fr.1-5 (A-E) at 220 nm.

Names	total	Compounds
50E 95E EE ME PE	2	19 43
95E EE ME PE	1	36
50E 95E ME PE	3	18 2 1
95E EE PE	1	42
95E ME PE	8	10 11 29 40 14 3 8 4
EE PE	18	33 7 26 27 20 31 12 15 45 30 25 28 24 22 46 23 13 34
ME PE	1	39
95E PE	1	6
95E ME	3	63 64 62
PE	11	32 21 16 44 35 41 17 9 38 37 5
EE	15	55 57 61 48 50 58 52 60 56 54 59 49 53 51 47
95E	3	65 66 67
50E	2	68 69

Figure S3. Outputs of venn diagram using the compounds 1-69 identified from PE, EE, ME, 95E, 50E by MS/MS-based spectral matching. The compound names have been shown in Table 5, and visualization of the results is presented in Figure 7A.

Table S1. The relative content of the major compounds identified from PE and EE by comparing their respective peak area to the total areas.

**Compound_Names** (**MD**[a] **≥ 95%**)	**Relative Peak Area** (%)				
	PE-Fr.1	PE-Fr.2	PE-Fr.3	PE-Fr.4	EE
Z-9-hexadecenoic acid	-[d]	32.69	39.40	-	36.06
Z-11-hexadecenoic acid	29.86	-	-	-	-
n-hexadecanoic acid	-	1.85	5.34	-	3.20
Ethyl 9-hexadecenoate	5.3	0.38	1.84	-	-
(Z,Z)-9,12-octadecadienoic acid	3.98	7.81	-	-	3.44
E-13-octadecenoic acid	12.83	-	-	-	-
6-octadecenoic acid	9.00	23.62	32.88	-	1.95
Ethyl oleate	4.58	2.58	-	-	-
Cholesterol	6.52	17.12	-	-	-
Unsaturated fatty acids	55.67	65.97	77.62	-	44.65
SUM[b]	68.09	86.05	79.42	0	44.65
SUM[C]	79.99	89.60	80.13	0	56.31
Unidentified peaks	20.01	11.40	19.87	100	46.69

Notes: a, MD means Matching Degree; b, SUM represents the sum of relative peak area of the compounds listed in this table; c, SUM means the total sum of relative peak area of all compounds identified by GC-MS with MD ≥ 95%; d, not detected.

Table S2. Compounds putatively identified from *Logia* extracts by MS/MS spectral comparison with cosine score value of 0.70

Compounds	RT_Query(s)	Precursor_MZ(Da)	Compound_Name	SharedPeaks	MQScore
**1**	70.802	166.087	Massbank:PB000408 Phenylalanine|2-amino-3-phenylpropanoic acid	6	0.956563
	70.802	166.083	Spectral Match to DL-Phenylalanine from NIST14	17	0.926237
**2**	82.189	205.098	Massbank:PB006083 Tryptophan|(2S)-2-amino-3-(1H-indol-3-yl)propanoic acid	25	0.954918
	82.189	205.097	Spectral Match to L-Tryptophan from NIST14	30	0.952962
**3**	83.523	188.07	Spectral Match to Abrine from NIST14(3-(2-Indolyl)-2-methylaminopropanoic acid)	10	0.920207
**4**	83.523	188.071	Spectral Match to DL-Indole-3-lactic acid from NIST14	13	0.921548
**5**	96.254	265.154	Spectral Match to Phe-Val from NIST14	19	0.787928
**6**	127.29	231.114	1,2,3,4-tetrahydroharmane-3-carboxylic acid	10	0.85109
**7**	147.378	136.076	Spectral Match to DL-Octopamine from NIST14	6	0.728619
**8**	151.331	279.17	Spectral Match to Phe-Leu from NIST14	14	0.923904
**9**	171.655	279.169	Spectral Match to Leu-Phe from NIST14	7	0.718983
**10**	214.147	279.169	Spectral Match to Phe-Ile from NIST14	8	0.909698
**11**	260.648	208.097	NCGC00042134-05_C11H13NO3_N-Acetylphenylalanine	12	0.836929
	260.648	208.097	Spectral Match to L-Phenylalanine, N-acetyl- from NIST14	10	0.91052
**12**	287.152	245.128	NCGC00380633-02_C14H16N2O2_Pyrrolo[1,2-a]pyrazine-1,4-dione, hexahydro-3-(phenylmethyl)-	18	0.718626
**13**	287.152	245.128	NCGC00381359-01_C14H18N2O3_Phenylalanine, prolyl-	19	0.747031
**14**	292.27	313.155	Spectral Match to Phe-Phe from NIST14	9	0.95749
**15**	393.38	164.107	N-acetyl-2-phenylethylamine	7	0.8942

**16**	883.698	333.206	NCGC00385270-01_C20H28O4_5-[2-(3-Furyl)ethyl]-8-hydroxy-5,6,8a-trimethyl-3,4,4a,5,6,7,8,8a-octahydro-1-naphthalenecarboxylic acid	168	0.727051
**17**	883.698	333.206	NCGC00385272-01_C20H28O4_5-[2-(3-Furyl)ethyl]-8a-(hydroxymethyl)-5,6-dimethyl-3,4,4a,5,6,7,8,8a-octahydro-1-naphthalenecarboxylic acid	169	0.730714
**18**	1018.02	415.211	NCGC00347704-02_C24H32O7_2H-Oxireno[1,10a]phenanthro[3,2-b]furan-10(11bH)-one, 5,7-bis(acetyloxy)-3,3a,4,5,6,7,7a,7b,8,8a-decahydro-4,4,7a,11-tetramethyl-, (1aS,3aR,5S,7S,7aR,7bS,8aR,11bR)-	49	0.887886
**19**	1018.02	415.211	NCGC00385811-01!6-[3-[(3,4-dimethoxyphenyl)methyl]-4-methoxy-2-(methoxymethyl)butyl]-4-methoxy-1,3-benzodioxole	25	0.896878
**20**	1101.95	301.215	Spectral Match to 14(15)-EpETE from NIST14	147	0.808564
**21**	1135.87	293.211	Spectral Match to 9(S)-HpOTrE from NIST14	52	0.712923
**22**	1148.93	301.216	Spectral Match to 17(18)-EpETE from NIST14	127	0.809739
**23**	1148.93	301.216	Spectral Match to (.+/-.)-8-Hydroxy-5Z,9E,11Z,14Z,17Z-eicosapentaenoic acid from NIST14	134	0.814645
**24**	1170.15	303.231	Spectral Match to 11S-Hydroxy-5Z,8Z,12E,14Z-eicosatetraenoic acid from NIST14	150	0.852725
**25**	1170.15	303.231	Spectral Match to 15(S)-Hydroxy-(5Z,8Z,11Z,13E)-eicosatetraenoic acid from NIST14	150	0.865231
**26**	1172.17	279.231	Spectral Match to Pinolenic acid from NIST14	96	0.773502
**27**	1176.45	295.226	Spectral Match to 13-Keto-9Z,11E-octadecadienoic acid from NIST14	86	0.820771
**28**	1178.25	277.216	Spectral Match to 13S-Hydroxy-9Z,11E,15Z-octadecatrienoic acid from NIST14	68	0.76488
**29**	1188.09	482.36	Spectral Match to 1-Hexadecyl-sn-glycero-3-phosphocholine from NIST14	7	0.887771

**30**	1191.89	317.211	9-hydroxy-1,4a-dimethyl-7-propan-2-yl-2,3,4,9,10,10a-hexahydrophenanthrene-1-carboxylic acid	114	0.732695
**31**	1191.89	317.211	NCGC00380535-01_C20H28O3_12-Oxopimara-9(11),15-dien-18-oic acid	132	0.727094
**32**	1200.88	277.216	Spectral Match to 9,12-Octadecadiynoic Acid from NIST14	63	0.707527
**33**	1211.54	317.211	7-ethenyl-1,4a,7-trimethyl-6-oxo-2,3,4,8,8a,9,10,10a-octahydrophenanthrene-1-carboxylic acid	51	0.713914
**34**	1216.52	295.227	Spectral Match to 9-Oxo-10E,12Z-octadecadienoic acid from NIST14	97	0.798115
**35**	1222.44	303.232	Spectral Match to 8S-Hydroxy-5Z,9E,11Z,14Z-eicosatetraenoic acid from NIST14	90	0.75798
**36**	1227.3	279.159	Spectral Match to Dibutyl phthalate from NIST14	9	0.923685
**37**	1290.23	323.258	Eicosanoids_15-oxoEDE_C20H34O3	45	0.722368
	1290.23	323.258	Spectral Match to 15-OxoEDE from NIST14	54	0.727381
**38**	1293.26	323.258	NCGC00169469-03_C20H34O3_1-Naphthalenecarboxylic acid, decahydro-5-(5-hydroxy-3-methylpentyl)-1,4a-dimethyl-6-methylene-, (1R,4aS,5R,8aS)-	107	0.736152
**39**	1316.52	552.401	Spectral Match to 1-Arachidoyl-2-hydroxy-sn-glycero-3-phosphocholine from NIST14	15	0.854794
**40**	1318.46	510.391	Spectral Match to Lyso-PAF C-18 from NIST14	9	0.893974
**35**	1349.1	303.23	Spectral Match to 8-HETE from NIST14	56	0.746991
**41**	1370.87	307.263	Spectral Match to Linolenic acid ethyl ester from NIST14	64	0.809892
**42**	1389.49	402.301	(Z)-N-hexadec-9-enoyl-L-phenylalanine	17	0.878151
**43**	1408.8	282.279	Spectral Match to 9-Octadecenamide, (Z)- from NIST14	37	0.795687
**44**	1463.23	404.316	NCGC00380823-01!2-(14-methylpentadecanoylamino)-3-phenylpropanoic acid	26	0.90229
**45**	1866.48	369.351	Spectral Match to Cholestan-3-one, (5.alpha.)- from NIST14	27	0.75334

**46**	1866.48	369.352	Spectral Match to Cholesterol from NIST14	24	0.763294
**47**	89.587	261.123	NCGC00384639-01_C14H16N2O3_Pyrrolo[1,2-a]pyrazine-1,4-dione, hexahydro-3-[(4-hydroxyphenyl)methyl]-	53	0.734621
**48**	115.327	180.102	N-acetyltyramine	9	0.811762
**49**	334.623	197.117	Loliolide	38	0.765238
	334.623	197.117	NCGC00385365-01_C11H16O3_2(4H)-Benzofuranone, 5,6,7,7a-tetrahydro-6-hydroxy-4,4,7a-trimethyl-, (6S,7aR)-	60	0.790237
**50**	354.718	146.06	Spectral Match to 1H-Indole-4-carboxaldehyde from NIST14	6	0.84395
**51**	356.703	284.139	cyclo(D-Trp-L-Pro)	16	0.950215
	356.703	284.139	NCGC00169940-02!3-(1H-indol-3-ylmethyl)-2,3,6,7,8,8a-hexahydropyrrolo[1,2-a]pyrazine-1,4-dione	17	0.933498
**52**	629.326	261.159	cyclo(Phe-Leu)	34	0.737895
**53**	662.681	334.155	NCGC00347762-02!3-benzyl-6-(1H-indol-3-ylmethyl)piperazine-2,5-dione	13	0.888409
**54**	684.967	295.129	Massbank:EA277006 Aspartame|3-amino-4-[(1-benzyl-2-keto-2-methoxy-ethyl)amino]-4-keto-butyric acid	11	0.751393
	684.967	295.129	Massbank:EA277012 Aspartame|3-amino-4-[(1-benzyl-2-keto-2-methoxy-ethyl)amino]-4-keto-butyric acid	14	0.750524
**55**	970.585	321.242	5-(1,2,4a,5-tetramethyl-7-oxo-3,4,8,8a-tetrahydro-2H-naphthalen-1-yl)-3-methylpentanoic acid	143	0.800957
	970.585	321.242	NCGC00180747-02!5-[(1S,2R,4aR)-1,2,4a,5-tetramethyl-7-oxo-3,4,8,8a-tetrahydro-2H-naphthalen-1-yl]-3-methylpentanoic acid	152	0.804541
**56**	1187.73	327.231	Spectral Match to (.+/-.)-11-Hydroxy-4Z,7Z,9E,13Z,16Z,19Z-docosahexaenoic acid from NIST14	82	0.733423
**57**	1187.73	327.23	Spectral Match to 19(20)-EpDPE from NIST14	80	0.725253

**58**	1261.82	297.242	Spectral Match to 9(10)-EpOME from NIST14	36	0.702697
**59**	1322.28	304.26	Spectral Match to Arachidonoyl amide from NIST14	74	0.857892
**60**	1471.59	358.31	phenylethylamide 357	46	0.81775
**61**	1551.27	628.187	NCGC00385199-01!2-[3,4-bis[[(2S,3R,4S,5S,6R)-3,4,5-trihydroxy-6-(hydroxymethyl)oxan-2-yl]oxy]phenyl]-5,7-dihydroxychromen-4-one	28	0.741822
**62**	132.068	263.138	Spectral Match to Phe-Pro from NIST14	14	0.772827
**63**	386.204	352.165	Spectral Match to Phe-Trp from NIST14	9	0.880003
**64**	506.815	352.165	Spectral Match to Trp-Phe from NIST14	9	0.815122
**65**	75.058	229.16	Spectral Match to Leu-Pro from NIST14	6	0.792858
**66**	200.695	332.218	Spectral Match to Thr-Val-Leu from NIST14	7	0.732416
**67**	207.742	277.119	Spectral Match to PyroGlu-Phe from NIST14	16	0.73009
**68**	62.857	182.081	Spectral Match to L-Tyrosine from NIST14	11	0.947038
**69**	99.71	295.128	Spectral Match to Glu Phe from METLIN	9	0.777446
